# Comparison of Two Radiofrequency Vessel-Sealing Device for Laparoscopic Ovariectomy in African Lionesses (Panthera Leo)

**DOI:** 10.3390/ani12182308

**Published:** 2022-09-06

**Authors:** Luca Lacitignola, Pietro Laricchiuta, Marta Guadalupi, Marzia Stabile, Annalaura Scardia, Mario Cinone, Francesco Staffieri

**Affiliations:** 1 Dipartimento Dell’Emergenze e Trapianti di Organo, sez. Cliniche Veterinarie e P.A., Università Degli Studi di Bari, s.p. per Casamassima km.3, Valenzano, 70010 Bari, Italy; 2 Zoosafari di Fasano, 72015 Brindisi, Italy; 3 Dottorato di Ricerca in “Trapianti di Tessuti ed Organi e Terapie Cellulari”, Università Degli Studi di Bari, 70100 Bari, Italy

**Keywords:** laparoscopy, ovariectomy, lionesses, LigaSure, Caiman 12, wound retractor laparoscopic device, Panthera Leo

## Abstract

**Simple Summary:**

This study evaluated two vessel-sealing devices for dissecting ovaries in adult obese African lionesses undergoing laparoscopic ovariectomy. We evaluated the surgery time the intra- and postoperative complications. The results of our study confirmed the significant advantages of employing the Caiman 12 vessel-sealing device in comparison with the LigaSure Atlas in terms of the time needed to complete ovariectomy, although both instruments could be considered safe. The use of the Caiman 12 is recommended when performing laparoscopic ovariectomies in adults with obesity.

**Abstract:**

To evaluate two vessel-sealing devices with different jaw lengths for dissecting ovaries in adult obese African lionesses undergoing laparoscopic ovariectomy. Twelve lionesses (*n* = 12) were recruited. The surgical procedures were performed through three portals, with a retractor platform positioned at the umbilical port and cannulas placed 3–4 cm cranial and caudal to the device at the level of the midline. Ovariectomy was performed using a vessel-sealing device according to a randomization list. We evaluated the surgery time the intra- and postoperative complications. The total surgery time was 49.3 min (range 40–61 min) in the Atlas group and significantly lower in the Caiman group (mean 31.8 min, range 26–51 min). The installation phase was similar between the groups. The ovariectomy time was significantly lower in the Caiman group (mean 7.8 min, range 4–11 min) than in the Atlas group (mean 20 min, range 16–30 min). Controlled bleeding was observed at the tip of the uterine horn in two cases in the Atlas group. No other complications were noted. The results of our study confirmed the significant advantages of employing the Caiman 12 vessel-sealing device in comparison with the LigaSure Atlas in terms of the time needed to complete ovariectomy, although both instruments could be considered safe. The use of the Caiman 12 is recommended when performing laparoscopic ovariectomies in adults with obesity.

## 1. Introduction

Laparoscopic ovariectomy (OVE) has been described successfully in many large felids and is preferred to open surgery because large felids have a very deep and wide abdominal cavity. This makes it difficult to visualize and ligate the mesovarium, even with a large ventral midline incision [1,2], in an effort to reduce the risk of suture line dehiscence and self-induced trauma, shorten recovery times, and reduce post-operative [1,3,4].

Laparoscopic OVE has been successfully described using three portals [3,5,6] or a single portal technique [2,7,8]. Both of these techniques use three instruments, which eliminates the need for transabdominal ovarian pedicle suspension to the abdominal wall, which is required when performing a two-cannula ovariectomy, as described for dogs and cats [9]. Although many techniques for ovarian pedicle ligation/dissection have been described in small animals, such as external Roeder’s knot, laser, harmonic scalpel, and radiofrequency bipolar vessel-sealing (RFVS) devices [10,11,12,13], in large felids, the use of RFVS devices [1,2,5,6,8,14] and harmonic scalpel [3] has been the only technique described for ovariectomy. Many authors, however, have found difficulties in cutting the cranial tip of the uterine horn, particularly in heavy adult or obese lionesses. The primary problems that occurred were multiple coagulation cycles and insufficient hemostasis that needed to be managed and monitored, resulting in surgical time extension [8]. Moreover, another important issue discussed in the previous studies was the amount of fat in the subcutaneous tissue and falciform ligament that provided elongation of surgical time when laparoscopic OVE has been performed in adult obese patients in comparison with cubs or normal scored body condition [2,6,8]. The advantages of using the wound retractor (WRD) port system have been recently described in obese lionesses [15] and horses [16], consisting of surgical time saving, simple extraction of the ovaries and the possibility of interrupting and re-establishing the pneumoperitoneum.

We hypothesized that employing an RFVS device with long jaws and more even distribution of the clamp pressure would decrease intraoperative complications and surgical time. As a result, the purpose of this study was to evaluate two RFVS devices with different jaw lengths for dissecting ovaries in adult obese African lionesses undergoing laparoscopic OVE.

## 2. Materials and Methods

### 2.1. Animal Welfare

Written informed consent was obtained for the study from the zoo (Leo 3000 S. p.a, c/o Zoosafari di Fasano, Brindisi, Italy). The surgical procedures were performed by an experienced veterinary surgeon (L. L.) and zoo veterinary team. The study was approved by the Ethical Committee for Clinical studies of the Dipartimento dell’Emergenze e Trapianti di Organi of Università degli Studi di Bari, “Aldo Moro” (Approval no. 3/2022). The study was conducted from February 2022 to March 2022.

### 2.2. Inclusion Criteria

The current study included animals participating in a birth control program chosen by the zoo’s owner and zoo’s vet (P. L.) to maintain a healthy population size, as specified by the European Association of Zoos and Aquaria recommendations for lion care guidelines. Adult (aged > 4 years) lionesses (Panthera Leo) were eligible for laparoscopic OVE; subadults were excluded from this study. Before surgery, abdominal ultrasonography was performed to evaluate the reproductive tract and presence of pregnancy. Pregnant animals and those with uterine disorders were excluded from the sterilization program.

### 2.3. Instrumentation and Grouping

**Figure 1:**
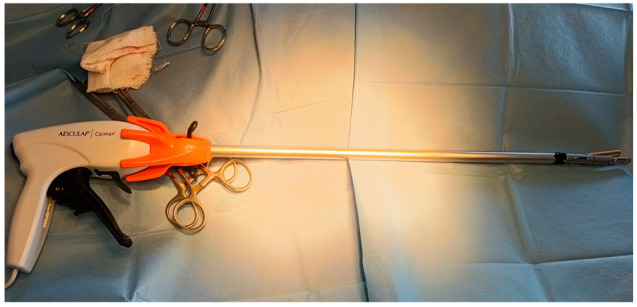
Image of the Caiman 12. The handpiece has a 12-mm wide diameter, with 50-mm long branches. The tip of the instrument is articulated.

Two radiofrequency vessel-sealing devices were used in this study and assigned to two different groups. In the group LigaSure, a 10-mm width RFVS devices with 21.4-mm long jaws (LigaSure Atlas, LS1037, LigaSure Atlas™ Tissue Fusion Laparoscopic Instrument, 37 cm, Medtronic, Milan, Italy) was employed. The handpiece was connected to a generator (ForceTriad, Medtronic, Milan, Italy), and the power was set to three bars. In the Caiman group, the jaws were 12-mm wide and 50-mm long (Caiman^®^ 12 articulated; Aesculap AG, Tuttlingen, Germany). The Caiman 12 handpiece was connected to the generator and the power was set at standard value (Figure 1). Although both handpieces were designed for a single use, the instruments were disposed of every two surgeries and sterilized in EtOx for reuse. Lionesses were assigned to a specific group according to a randomization list obtained from randomization online software (www.randomizer.org, accessed on 15 January 2022).

### 2.4. Capture and Anesthesia

With the help of zookeepers, eligible animals were isolated from the whole group in a separate environment the day before the planned surgery. Each animal was individually recognized using a unique number of microchips. The animals were fasted for 8 h and water was withheld for 3 h before surgery. The animals were darted with a combination of detomidine 0.05 mg/kg and ketamine 2 mg/kg. After complete immobilization, the lionesses were transported to the operating theatre. A 14-gauge intravenous cannula was placed in the cephalic vein, and propofol (1–2 mg/kg) was administered to allow placement of an orotracheal tube with an 18-mm ID. Anesthesia monitoring included heart rate, non-invasive arterial blood pressure, oxygen saturation (SpO2), capnography, and body temperature. The animals were maintained on isoflurane in pure oxygen during surgery with spontaneous ventilation. A single dose of methadone (10 mg) was administered to all the animals. In case of sudden variations in HR, respiratory rate, and blood pressure higher than 20%, a dose of fentanyl 2 µg/kg intravenously was considered as rescue analgesia. Cefazoline 20 mg/kg and meloxicam 0.2 mg/kg were administered intravenously within 30 min of anesthesia induction. At the end of the procedure, isoflurane was discontinued, and the animals were moved to the recovery area with proper assistance. For anesthetic reversal, atipamezole was administered i.m. at 5-fold the detomidine dose.

### 2.5. Surgical Procedure

The lionesses were positioned in the dorsal recumbency on a custom-made electrical tiltable table. The abdomen was clipped and aseptically prepared for the surgery.

A wound retractor port system (Endo Keeper model CG sized 265-mm length × 95-mm large Nelis, Bucheon Techno Park, Ssangyong, Korea) was placed 1 cm caudal to the umbilicus as the first port according to a technique described previously [15].

**Figure 2:**
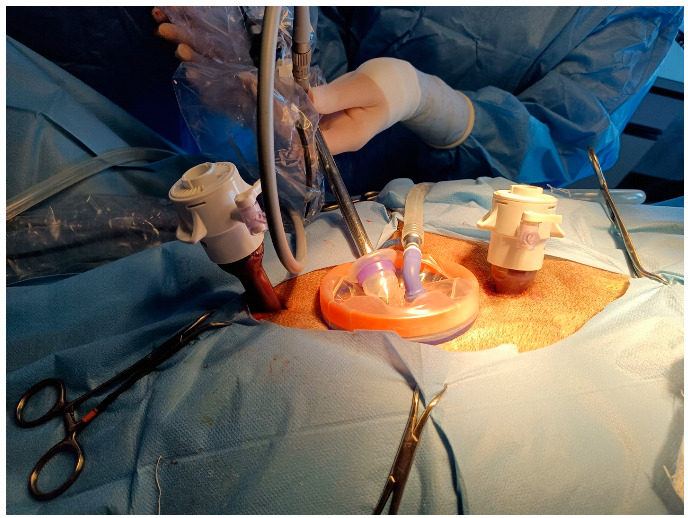
Portal positioning. Cranial is on the right. WRD was placed at 1 cm caudal to the umbilicus. The cranial and caudal 12-mm cannulas were placed in the midline 3–4 cm from WRD border.

The laparoscope (10-mm diameter; 30° angle of vision, HOPKINS II, Karl Storz Endoskope GMBH & Co. KG; Tuttlingen, Germany) was placed in the abdominal cavity. The other two portals were created using two laparoscopic cannulas with a blunt trocar (12-mm diameter; 20-cm length; Applied Medical, Milan, Italy) positioned on the midline, 3–4 cm cranial and caudal to the ring, under laparoscopic visualization (Figure 2).

Complications included injury to the abdominal organs. The table was tilted by 45° with the lionesses in the right lateral recumbency, and the grasping forceps were introduced into the cranial cannula and the laparoscope into the central port cannula. The ovary was suspended, and the ovarian pedicle, proper ligament, and suspensory ligament were coagulated and transected using a laparoscopic vessel-sealing device (assigned to the group) placed in the caudal port. After complete dissection, the telescope was moved to the caudal port, and 10-mm grasping forceps were introduced to grasp the ovary. The cap was removed from the platform and the ovaries were retrieved through the wound retractor port. After re-establishing the pneumoperitoneum and repositioning the lionesses in opposite recumbency, the right ovariectomy was performed in the same manner as the left one. After the second ovary was retrieved, the lioness was repositioned in dorsal recumbency, the retractor was removed, and the external and internal rings were removed by pulling the removal tag. The umbilical portal was closed with two layers of PDS (0-USP; Ethicon, Milan, Italy) with interrupted mattress sutures. The 12-mm portals were sutured in one-layer PDS (0-USP; Ethicon, Milan, Italy) with a single interrupted mattress suture.

### 2.6. Clinical and Surgical Variables

Weight (kg ± standard deviation [SD]) was recorded. The body condition score was determined by visual inspection according to a previously described scale [17]. The estrus status was determined by hormone levels and colocalization.

The evaluated surgical variables were the total surgery time (min, mean ± SD, range) from the first skin incision to the last suture placement, the laparoscopic portal installation time (min, mean ± SD, range) from the first skin incision to the last cannula placement, and the ovariectomy time (min, mean ± SD, range) from the last trocar placement to the second resected ovary retrieval.

Moreover, the number of attempts to correct the cannula placement, inadvertent abdominal organ injury, and intraoperative complications were recorded. Bleeding was scored according to a semiquantitative score assigned by the primary surgeon according to the following criteria: 0 = no bleeding; 1 = mild bleeding (a few drops, self-limiting, without any influence on visibility); 2 = moderate bleeding (bleeding with little influence on visibility, requiring additional application of the vessel-sealing device); 3 = severe bleeding (with a need for conversion to open surgery and hemostasis). The number of coagulation cycles for ovarian resection was calculated retrospectively by watching the video recorded during surgery.

Overall, tissue specimens from the ovaries and uterus attachment diameters were measured with a caliber after removal (mm, mean ± SD, range). The ovaries were used in additional studies.

### 2.7. Postoperative Regimen and Aftercare

After extubation, the animals were placed in a cage in a calm environment and isolated from other individuals to allow recovery from anesthesia. Once fully recovered, the lesions were observed for variation in behavior, feeding, signs of pain and distress, and wound dehiscence and bleeding. Twenty-four hours after recovery, the animals were released into the normal environment. To prevent further pharmacological restrictions in the early follow-up, a post-surgical examination was scheduled only in cases of lack of appetite, bleeding from surgical incisions, or clear evidence of hernia. Long-term follow-up was scheduled 4–5 weeks after surgery.

### 2.8. Statistical Analysis

Parametric data are expressed as the mean ± standard deviation and range. Non-parametric data are expressed as the median ± standard error and range. Significant differences were calculated by one-way analysis of variance for parametric data and Kruskal–Wallis test for non-parametric data, performed with “The jamovi project (2021). jamovi. (Version 2.2) [Computer Software], retrieved from https://www.jamovi.org”(accessed on 30 March 2022). The significance level was set at *p* < 0.05.

## 3. Results

### 3.1. Clinical Study Population

Twelve lionesses were selected from the zoo’s property and vetted for inclusion in the sterilization program because they were not intended to be bred. However, during the presurgical ultrasound examination, a pregnant animal was originally assigned to the Caiman group and excluded from the surgical procedures. Therefore, six (*n* = 6) and five (*n* = 5) lionesses were included in the Atlas and Caiman groups, respectively. Table 1 summarizes the mean, median, standard deviation, and range for the weight and BCS. No statistically significant differences were detected between the groups (*p* > 0.05) for weight and BCS. In the Atlas group, three lionesses were in proestrus (*n* = 3), two were in diestrus (*n* = 2), and one was in estrus (*n* = 1). In the Caiman group, no lioness was observed in the proestrus (*n* = 0), three in diestrus (*n* = 3), and two in estrus (*n* = 2).

### 3.2. Surgery

Laparoscopic OVE was completed in all patients, without conversion to open surgery. Rescue analgesia was not administered to any of the animals. The installation phase, ovariectomy, and total surgery times are summarized in Table 1. No significant difference was detected between the groups during the installation phase (*p* > 0.05). The WRD was inserted through a 25-mm mini-laparotomy port. Nonetheless, the considerable amount of subcutaneous fat necessitated meticulous dissection to reach the linea alba. Even after access to the abdominal cavity, an excessive amount of falciform ligament fat requires special attention to the insertion of the WRD inner ring. However, when the ring was properly placed, the fat was pushed away and optimum wound retraction was achieved in all cases. Although the subcutaneous amount of fat made the linea alba difficult to view, the insertion of the caudal cannula was not problematic. The cranial cannula placement, conversely, was more difficult due to the significant fat in the falciform ligament, which required the cannula to penetrate through the ligament in certain cases for proper placement. No cases required re-insertion of cannulas in the first attempt, and no entry-related complications were observed. The cannula location allowed optimal triangulation with good organ manipulation without interference from the instruments. The use of a motorized custom-made surgical table eased the tilting maneuvers necessary for ovary vision and manipulation at a 45° angle.

**Figure 3:**
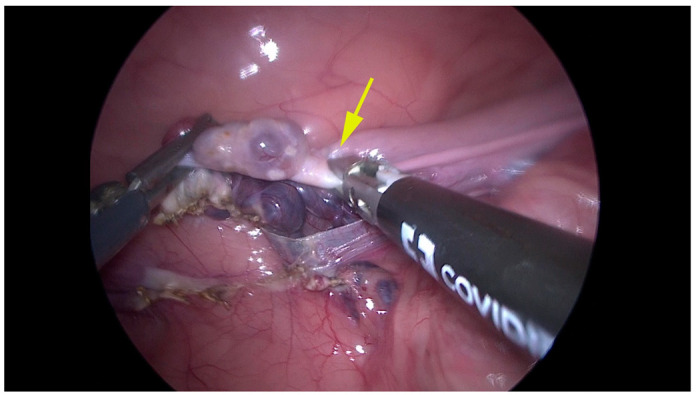
Representative image of the right ovariectomy performed by Atlas. Cranial is on the left. Yellow arrow shows that the branches clamped incompletely the cranial tip of the uterus incompletely, requiring several applications to be completed.

The ovariectomy time was significantly shorter (*p* < 0.05) in the Caiman group (7.80 min; range 4–11 min) vs. the Atlas group (20.0 min; range 16–30 min). The coagulation of ovarian vessels was completed correctly with the aid of both vessel-sealing devices, and dissection of the proper ligament and mesovarium was conducted without difficulty. The dissection at the proper ligament in some cases included the cranial uterine horn (4/6 in the Atlas group; 4/5 in the Caiman group), necessitating many applications of the vessel-sealing device in the Atlas group. Moreover, in the Atlas group, we noticed tissue sliding from the jaws of the instrument and poor coagulation, necessitating additional coagulation cycles (Figure 3).

**Figure 4:**
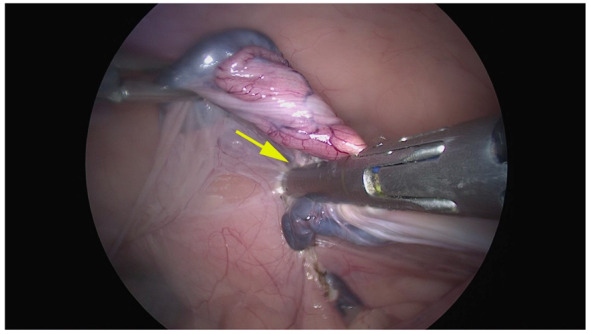
Representative image of right ovariectomy performed by the Caiman 12. The yellow arrow shows that the entire tissue was completely included between the branches.

In the Atlas group, we noticed a grade 1 bleeding score in one case and grade 2 hemorrhage from the cranial uterine horn in two cases, which necessitated several coagulations and bleeding monitoring. However, the bleeding was successfully managed and did not necessitate conversion. In the Caiman group, no bleeding was observed (median score = 0 ± s. e. 0, range 0), and the median bleeding score in the Atlas group was 0.5 ± s. e. 0.4; range 0–2). However, this difference was not statistically significant (*p* > 0.05) (Figure 4).

The mean number of coagulation cycles for resection of the proper ligament of the cranial tip or the uterine horn mean was 10.8 cycles (± S.D. 11.1; range 2–40) in the Atlas group and significantly (*p* < 0.05) lower for the Caiman group (mean 1 cycle; S.D. 0; range 1–1).

The ease with which the platform cap could be removed and reinserted during the ovarian extraction maneuver allowed the pneumoperitoneum to be restored to the correct pressure without the need for platform repositioning. The ovary retrieval procedure was simple, with a smooth passage from the retractor and no need to widen the portal. The mean size of the entire specimens was 42.1-mm length (S.D. 14.4 mm; range 24.8–61.9 mm) and 30.7-mm width (S.D. 12.6; range 14.7–46.1 mm). The uterus (measured on the specimens) mean diameter was 14.3 mm in the Atlas group (S.D. 5.28 mm; range 10–29 mm) and 25.9 mm in the Caiman group (S.D. 9.48 mm; range 11–36 mm), which was significantly thicker (*p* > 0.05).

**Figure 5:**
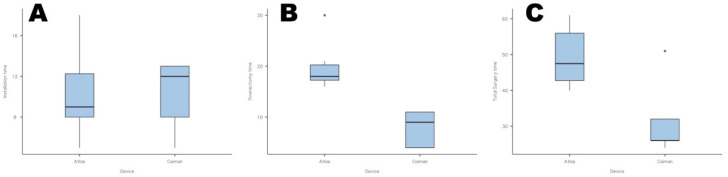
Box plots representing the installation time (**A**), ovariectomy time (**B**), and total surgery time (**C**), respectively for the Atlas and Caiman groups. Statistical differences (*p* < 0.05) were observed or the ovariectomy time and total surgery time. Dots represent outliers.

The mean total surgery time for the Atlas group was 49.33 min (S.D. 8.6; range 40–61 min). Conversely, in the Caiman group, the mean time to complete surgery was significantly lower (*p* < 0.05), with 31.8 (S.D. 11.1 min; range 26–51 min) (Figure 5; Table 1).

### 3.3. Post-Operative Evaluation

All animals recovered from anesthesia without any issues and were returned to the rest of the group after 24 h.

Postoperatively, none of the animals showed signs of anorexia, reluctance to move, isolation, or altered social behavior. Unfortunately, physical clinical examinations were not performed for all operated animals in the early postoperative period to prevent early re-capture or physical and chemical restraints, which might cause further stress to the animals. However, clinical examination was performed under sedation 5 weeks post-operative, with the results indicating there was no evidence of surgical site infection, dehiscence, or hernia at the portal sites after a follow-up.

## 4. Discussion

In this study, we investigated the surgical time and immediate perioperative complications of LigaSure Atlas versus Caiman vessel-sealing devices in patients undergoing laparoscopic OVE. The results obtained in the current study showed that the Atlas and Caiman devices safely sealed the ovarian vessels and performed complete dissection of the proper ovarian ligament, suspensory ligament, and mesovarium. However, the Atlas device necessitated several coagulation cycles to complete dissection of the ovary, especially when the tip of the uterine horn was inadvertently included. Furthermore, when using the Atlas, we observed moderate bleeding from the uterus in two cases. Conversely, the Caiman performed complete sealing in all cases, without any bleeding. Moreover, the Caiman device significantly reduced the number of coagulation cycles and the time to complete ovariectomy, avoiding overheating effects on tissues that has been described to have a negative impact on seal quality [17] and prolong surgery. In fact, in the Caiman group, we observed a significant reduction in ovariectomy time, resulting in a reduction in the total time needed to complete the surgery. We noticed that the ovariectomy time was the phase of the surgical procedure that was considered the most time spent. In fact, no significant differences were observed between the groups in the installation of the portal phase and suture time. We suppose that the main advantages of the Caiman devices versus the Atlas were the longer jaws and more even pressure achieved on the tissue when clamped. In fact, the Caiman has 50-mm long jaws versus 21.4 mm of the Atlas. Longer jaws allow the inclusion of more tissue for every bite. Moreover, the specific design of the Caiman’s jaw allows a uniform pressure along the branches, performing a complete and uniform coagulation along the tissue [18]. This feature was also confirmed by the higher Caiman performance even if the uterus was significantly wider. In fact, although randomly assigned to each group, the uterus diameter was wider in the lionesses in which the Caiman device was employed than in the Atlas group. Unfortunately, the influence of the estrus status on bleeding, number of coagulation cycles, and time to complete ovariectomy was not considered in this study because the distribution of lionesses in different phases of estrus was not uniform between the groups. Although it has been reported that the estrus cycle has an effect on uterine texture in dogs [19], we subjectively appreciated that lionesses during proestrus had a hard uterus, resulting in more difficulty in clamping the tissue between branches. Furthermore, surgeons should keep in mind that the vessel-sealing devices employed in this study use an impedance feedback-controlled energy deployment algorithm created specifically for sealing vessels. This system is regulated by a tissue impedance reading of up to 100.000 reads per second, which modifies the necessary power and cycle time based on tissue impedance calculation by the generator. Correct sealing of the vessel is achieved by collagen and elastin denaturation during the coagulation cycle [20]. Although the anatomical structure and collagen and elastin contents of the uterus are completely different from those of vessels, the hemostatic effect on the uterus is obtained by the thermal effect of the coagulation cycles; however, sealing is not achieved constantly. Another important factor in obtaining a complete sealing of hollow organs is the compression of the tissue during the coagulation cycles [21]. In canine species, it has been reported that several coagulation cycles may affect the strength of the seal of the hollow organ as a consequence of the excess of thermal injury [19]. The tissue sealing has been shown to be influenced by the compressive pressure applied by the instrument’s jaws [18,20,21,22,23,24]. Both the instruments employed in the study deliver compression not controlled by the surgeon. The actual amount of compression is not stated by the manufacturer, but we can assume that the Caiman has a unique branch design and mechanism capable of applying a higher and more uniform force along the jaws, gripping the branch’s tip first [25]. The uniform compression along the jaws and their longer branches allowed better sealing performance in the lioness uterus. The diameter of the uterine horn in our study had a mean of 14.3 mm in the Atlas group and 25.4 mm in the Caiman group. It has been described that in other species, uteri wider than 9 mm could be not completely sealed with an Atlas device [19]. However, in another study in cats, uteri affected by pyometra with a diameter of >9 mm were effectively sealed with a 12-mm Caiman device [26]. The results of our study confirmed that the Caiman device can be safely employed to resect the uterine horn with a diameter up to ca. 36 mm. Dividing the tip of the uterine horn has no clinical consequences in dogs or cats [19].

The mean total surgery time was 49.33 min. The average time to finish surgery in the Caiman group was 33.75 min, which was significantly shorter (*p* < 0.05). Prior studies in which adult and obese lionesses were laparoscopically ovariectomized yielded longer surgeries [2,8]. These results can be influenced by several factors. In fact, it has been reported that the significant subcutaneous and falciform fat created difficulties in ovaries extraction [6]. For this reasons, we used a wound retractor laparoscopic platform at the umbilical portal as previously described [15] rather than the 11-mm cannula [3,4,6,8] or single-port multi-cannulated platform [2,7,8], reported in large felids. The WRD was placed via a 25-mm mini-laparotomy, which provided sufficient space for correct subcutaneous dissection to reach the linea alba for access to the peritoneal cavity. Furthermore, after the peritoneum has been incised, the falciform ligament may be readily dissected or pushed aside, and the WRD can be firmly secured to the abdominal wall, resulting in radial (360°) retraction on the mini-laparotomy.

Although the use of a single-port multi-cannulated laparoscopic port has previously been proposed in these species [2,6,8], the use of the platform described in other reports (SILS port) in obese adult lioness may encounter certain technical difficulties. In particular, the authors found instrument collisions and loss of triangulation in tigers and lionesses, resulting in surgical time lengthening [2,7,8].

The mean ovarian size was 42 × 30 mm. Although the ovaries were removed from the abdomen during ovariectomy, this procedure had little effect on this phase. In fact, even with large specimens, the application of WRD enabled the extraction of harvested ovaries directly via mini-laparotomy without difficulty. Radial retraction of the surgical site allowed the specimens to pass through, with minimal pressure and reduced tissue trauma. WRD implanted through a 25-mm mini-laparotomy eliminates the need for the incision to be enlarged. The plastic layer that covers the incisions protects the incision tissues and prevents dehydration while also providing a smooth surface that facilitates extraction. After ovary extraction, the WRD must be closed again to re-establish a firm seal for the pneumoperitoneum required for the second ovary removal, as described for other single-port platforms [8]. However, the platform cap is simply applied and removed, making the operation easy and time-saving, eliminating the need for suturing the portal incision to seal the door around the usual 10–12 mm cannula after difficult organ extraction.

There were no complications during the procedure or in the early post-operative period. Unfortunately, due to the aggressiveness and risks of large felids during management, direct examination of wounds was impossible soon after, and in accordance with the zoo property and zoo vets, we excluded further chemical restriction in the short-term follow-up, limiting post-operative evaluation from afar, and evaluation of variation in behavior and appetite, which represents an important limitation of this study. However, in accordance with zoo’s vet team, all lionesses were re-examined under sedation 5 weeks after surgery, showing no evidence of surgical site infection, dehiscence, or signs of hernia.

This study has several limitations. In particular, although the study population is limited to 11 subjects, it must be considered that our sample represents the largest with regard to the presence of only obese adult subjects, whereas in the limited number of studies in the literature the populations investigated included mainly young or sub-adult subjects. Unfortunately, it was not possible to expand the number of animals because gonadectomy represents a choice limited to subjects with poor genetics and other contraception techniques are preferred in young or valuable subjects. In addition, the presence of zoos where this species requires population control is limited in our country.

Another limitation is the uneven presence in the two groups of subjects at the same stage of the estrous cycle. Unfortunately, the evaluation of the estrous cycle was performed at the time of surgery and therefore evaluated a posteriori. We are aware that the estrous cycle affected the texture, stiffness, and blood supply of the uterus, and an assessment before surgery could have directed a more uniform distribution of the population between the groups. However, no subject was allowed to undergo clinical examinations and laboratory investigations without sedation, and the subjects were not trained in veterinary care. For these reasons, zoo ownership allowed such an evaluation only at the time of surgery to avoid exposing the animals to several anesthetic procedures.

## 5. Conclusions

The results of our study confirmed the significant advantages of employing the Caiman 12 vessel-sealing device in comparison with the LigaSure Atlas in terms of the time needed to complete ovariectomy although both instruments resulted safe. Therefore, we recommend the use of the Caiman 12 when performing laparoscopic ovariectomies in obese adult patients.

## Figures and Tables

**Table 1:** Summary of the mean, median, standard deviation, and range for the weight, BCS, installation time, ovariectomy time, and total surgery time. (*) significant differences detected (*p* < 0.05).

	Device	Weight (Kg)	BCS	Installation Time (min)	Ovariectomy Time (min)	Total Surgery Time (min)
Mean	Atlas	173	7.83	10.3	20.0	49.3
	Caiman	179	7.40	10.2	7.80 *	31.8 *
Median	Atlas	168	8.00	9.00	18.0	47.5
	Caiman	180	7.00	12.0	9.0	26.0
SD	Atlas	17.8	0.753	4.59	5.18	8.62
	Caiman	7.33	0.54	3.56	3.56	11.1
Minimum	Atlas	150	7.00	5.00	16.0	40.0
	Caiman	170	7.00	5.00	4.00	26.0
Maximum	Atlas	200	9.00	18.0	30.0	61.0
	Caiman	190	8.00	13.0	11.0	51.0

## Data Availability

All data presented in this study are available in the paper.
